# Automated analysis of free speech predicts psychosis onset in high-risk youths

**DOI:** 10.1038/npjschz.2015.30

**Published:** 2015-08-26

**Authors:** Gillinder Bedi, Facundo Carrillo, Guillermo A Cecchi, Diego Fernández Slezak, Mariano Sigman, Natália B Mota, Sidarta Ribeiro, Daniel C Javitt, Mauro Copelli, Cheryl M Corcoran

**Affiliations:** 1 Department of Psychiatry, College of Physicians and Surgeons of Columbia University, New York, NY, USA; 2 Division on Substance Abuse, New York State Psychiatric Institute, New York, NY, USA; 3 Department of computer Science, School of Sciences, Universidad de Buenos Aires, Buenos Aires, Argentina; 4 Computational Biology Center—Neuroscience, IBM T.J. Watson Research Center, Yorktown Heights, NY, USA; 5 Department of Physics, School of Sciences, Universidad de Buenos Aires, Buenos Aires, Argentina; 6 Brain Institute, Federal University of Rio Grande do Norte, Natal, Brazil; 7 Division of Experimental Therapeutics, New York State Psychiatric Institute, New York, NY, USA; 8 Department of Physics, Federal University of Pernambuco, Recife, Brazil

## Abstract

**Background/Objectives:**

Psychiatry lacks the objective clinical tests routinely used in other specializations. Novel computerized methods to characterize complex behaviors such as speech could be used to identify and predict psychiatric illness in individuals.

**AIMS:**

In this proof-of-principle study, our aim was to test automated speech analyses combined with Machine Learning to predict later psychosis onset in youths at clinical high-risk (CHR) for psychosis.

**Methods:**

Thirty-four CHR youths (11 females) had baseline interviews and were assessed quarterly for up to 2.5 years; five transitioned to psychosis. Using automated analysis, transcripts of interviews were evaluated for semantic and syntactic features predicting later psychosis onset. Speech features were fed into a convex hull classification algorithm with leave-one-subject-out cross-validation to assess their predictive value for psychosis outcome. The canonical correlation between the speech features and prodromal symptom ratings was computed.

**Results:**

Derived speech features included a Latent Semantic Analysis measure of semantic coherence and two syntactic markers of speech complexity: maximum phrase length and use of determiners (e.g., *which*). These speech features predicted later psychosis development with 100% accuracy, outperforming classification from clinical interviews. Speech features were significantly correlated with prodromal symptoms.

**Conclusions:**

Findings support the utility of automated speech analysis to measure subtle, clinically relevant mental state changes in emergent psychosis. Recent developments in computer science, including natural language processing, could provide the foundation for future development of objective clinical tests for psychiatry.

## Introduction

The capacity of psychiatry to diagnose and treat serious mental illness has been hampered by the absence of objective clinical tests of the type routinely used in other fields of medicine. Although recent years have seen substantial advances in understanding of the neurobiology of mental illness,^1^ these developments have yet to yield markers that reliably differentiate psychiatric health from illness at the level of the individual patient. Whereas clinical neuroscience has focused on the brain in mental illness, computer science has, in parallel, developed increasingly sophisticated automated approaches to characterize and predict human behavior. Such advances are now commonly utilized in industry (the private business sector): models combining demographic data and purchasing behavior are used to personalize advertising content^2^ and automated language assessment is employed to screen job candidates and score essays.^3^ The degree to which such technologies might also aid diagnosis and prognosis in psychiatry is only now beginning to be explored (e.g., see ref. 4).

Developments in automated natural language processing^5^ present one promising avenue for psychiatry. Although speech may present a unique ‘window into the mind’ in a variety of altered states,^6^ it is particularly relevant to psychosis. Thought disorder, a cardinal symptom of schizophrenia in which thought processes lose coherence, is typically diagnosed on the basis of clinical observation of disorganized speech.^7^ As a complement to clinical observation, automated analysis methods have previously been used to assess speech correlates of thought disorder in schizophrenia.^8^ For example, Latent Semantic Analysis (LSA), an automated high-dimensional associative analysis of semantic structure in speech, has been used to identify a reduction in semantic coherence in schizophrenia that correlates with clinical ratings and has comparable diagnostic accuracy.^3^ LSA combined with structural speech analysis was also able to accurately differentiate between first-degree relatives of schizophrenia patients and unrelated healthy individuals, suggesting that subtle differences indicative of underlying genetic vulnerabilities to schizophrenia can be distinguished with computerized speech analysis.^9^

As yet, however, these methods have not been applied to the prediction of psychosis onset, even though clinically diagnosed subtle disorganization in speech has consistently been identified as predictive of psychosis (i.e., with classification accuracy of ~60%) among young people identified as at clinical high risk (CHR) for psychosis (reviewed in ref. 10), as well as those at genetic high risk for psychosis.^11^ There are several reasons to test automated prediction approaches in this population. Schizophrenia, although relatively rare (lifetime prevalence ~1%), is among the most catastrophic mental illnesses both personally and societally. Schizophrenia and related psychotic illnesses typically emerge in young adults at the point of maximal societal and parental investment when individuals are poised to begin to contribute socially and economically.^12^ Although those at CHR for developing schizophrenia by virtue of subthreshold or attenuated psychotic symptoms can be identified,^13^ to date reliable prediction of psychosis onset among high-risk youths has proven elusive. Improving the capacity to predict psychosis among high-risk populations would have important ramifications for early identification and preventive intervention, potentially critically altering the long-term life trajectory of people with emergent psychotic disorders.

Here, we present a proof-of-principle test of automated speech analysis to predict, at the level of the individual, the later onset of psychosis. Specifically, we employed analysis of free speech at baseline to predict psychosis onset over a subsequent period of up to 2.5 years in teens and young adults identified as at CHR for psychosis.^13^ On the basis of earlier findings in schizophrenia,^3,9,14^ in which automated text analyses yielded parameters that accurately discriminated between patients and controls, we hypothesized that automated semantic and syntactic analysis of baseline interview transcripts would yield speech features capable of predicting subsequent psychosis outcome among CHR individuals.

## Materials and methods

### Participants

Participants were 34 help-seeking youths aged 14 to 27 years who were fluent in English (three were immigrants who learned English as children). They were referred from schools and clinicians, or self-referred through the Center of Prevention and Evaluation website. Exclusion criteria included history of threshold psychosis or Axis I psychotic disorder, risk of harm to self or others incommensurate with outpatient care, any major medical or neurological disorder, and Intelligence Quotient<70 (assessed with the Wechsler Abbreviated Scale of Intelligence). The attenuated psychotic symptoms characteristic of the CHR participants could not have occurred solely in the context of substance use or withdrawal. Adults provided written informed consent; participants under 18 provided written assent, with consent provided by a parent. All experiments were performed in accordance with the relevant guidelines and regulations, and all procedures were approved by the Institutional Review Board at the New York State Psychiatric Institute at Columbia University. Five participants transitioned to psychosis within 2.5 years of follow-up (CHR+), whereas 29 did not (CHR−). Demographics for CHR individuals, stratified by psychosis outcome, are presented in Table 1.

### Procedures

#### Ascertainment and prospective characterization

The Structured Interview for Prodromal Syndromes/Scale of Prodromal Symptoms (SIPS/SOPS)^13^ was used for ascertainment of CHR status, for baseline and quarterly symptom ratings,^10^ and to determine psychosis outcome. The SIPS/SOPS evaluates positive (subthreshold psychotic), negative, disorganized, and general symptoms.

Participants had to meet baseline criteria for one of three prodromal syndromes, assessed with the SIPS/SOPS: (i) attenuated positive symptom syndrome (⩾1 SOPS-positive item in the prodromal range with symptoms beginning or worsening in the past year, and symptoms occurring ⩾once/week in the prior month); (ii) genetic risk and deterioration syndrome (psychosis in a first-degree relative or schizotypal disorder accompanied by a 30% drop in global assessment of function over the past year); or (iii) brief intermittent psychotic symptom syndrome (⩾1 SOPS-positive items in the psychotic range with symptoms beginning in the past 3 months, and symptoms occurring ⩾several minutes/day). All CHR participants in this study met criteria for the attenuated positive symptom syndrome. Trained master-level research assistants administered the SIPS/SOPS, with clinical ratings achieved by expert consensus (with CC).

Participants were prospectively characterized for symptoms every 3 months for up to 2.5 years, with transition to psychosis determined using the SIPS/SOPS ‘presence of psychosis’ criteria.

#### Baseline interviews

Open-ended, narrative interviews of ~1 h were obtained from participants by interviewers trained by an expert in qualitative interviewing and phenomenological research.^15^ Participants were encouraged to describe changes they had experienced and the impact of these changes, what had been helpful or unhelpful for them, and their expectations for the future. Interviews took place between 2007 and early 2012, and were transcribed by an independent company. The first 27 transcripts were previously subject to thematic analysis using phenomenological procedures, finding gender differences in themes; this earlier qualitative analysis did not assess the predictive value of the interviews for psychosis outcome.^16^

#### Speech preprocessing

Interview transcripts were preprocessed as previously described^6^ using the Natural Language Toolkit (NLTK; http://www.nltk.org/).^5^ After discarding punctuation, each interview was automatically parsed into phrases. Words were then converted to the roots from which they are inflected, or lemmatized, using the NLTK WordNet lemmatizer. The resultant preprocessed data consisted of a list of lemmatized words, parsed into phrases, maintaining the original order, without punctuation and in lower case.

#### Speech analyses

We employed a novel combination of semantic coherence and syntactic assays as predictors of psychosis transition. For the semantic analyses, we used a well-validated approach to automated text analysis previously used to analyze speech in schizophrenia,^3^ LSA^17^. LSA is a high-dimensional associative model that rests on the premise that word meaning is a function of the relationship of each word to every other word in the lexicon. If semantically similar words co-occur in texts with consistent topics more frequently than do unrelated words, then the semantic similarity of two words can be quantitatively indexed by the frequency of their co-occurrence in a sufficiently large corpus of texts.^17^ LSA thus captures the meaning of words through linear representations in high-dimensional (300–400 dimensional) semantic space based on word co-occurrence frequencies. Each word in the lexicon is assigned a vector representing its semantic content; the orientation of these vectors can then be used to compare semantic similarity between words.^17^

**Figure 1:**
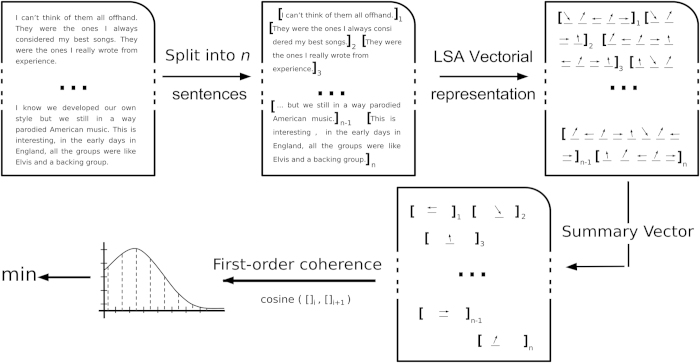
Pipeline for automated extraction of the semantic coherence features. Texts were initially split into sentences/phrases. Each word was represented as a vector in high-dimensional semantic space using Latent Semantic Analysis (LSA). Summary vectors were calculated as the mean of each vector in each phrase. Coherence was determined based on the semantic similarity between adjacent phrases, calculated as the cosine of their respective vectors. The semantic coherence feature that best discriminated those who transitioned to psychosis from those who did not was the minimum semantic coherence value (i.e., the coherence at the point of maximal discontinuity) within each transcribed text.

Here, LSA was trained on the Touchstone Applied Science Associates (TASA) Corpus, a collection of educational materials compiled by TASA. The semantic coherence measure we developed is similar to that used by Elvevåg *et al.*,^3^ which discriminated between established schizophrenia patients and controls. The present measure differs from the earlier approach in that it explicitly incorporates syntactic information: semantic trajectories are represented by similarity among pairs of consecutive phrases, or pairs of phrases separated by an intervening phrase (see Figure 1). Given the speech transcription *D*, the document is split into *n* phrases *S_i_* and converted into a vectorial representation by replacing each word in the phrase by its corresponding LSA vector, $S_{i}\to \left\{l_{i1},\vec{\leftarrow },l_{iN}\right\}$. The phrase vectors are then summarized by taking the mean of their components:

$$L_{i}=\frac{1}{N}\sum_{k=1}^{N}l_{ik}$$

i.e., the mean of all LSA vectors of every word in the phrase.

We defined first-order coherence by taking the similarity of consecutive phrase vectors, averaged over all the phrases in the text (represented by 〈.〉 below):

$$\mathrm{FOC}=\left\langle \cos\;(L_{i},L_{i+1})\right\rangle$$

and second-order coherence by taking the similarity between phrases separated by another intervening phrase, averaged over all the phrases in the text:

$$\mathrm{SOC}=\left\langle \cos(L_{i},L_{i+2})\right\rangle$$

With these two features, we were able to characterize semantic coherence by measuring components of the distributions of first- and second-order coherence over the speech samples, including features such as the minimum, mean, median, and s.d.

Thus, we indexed speech coherence by: (i) automated separation of interviews into phrases; (ii) assigning phrases semantic vectors as the mean of the LSA semantic vectors for each word within the phrase; and (iii) assessing semantic similarity (i.e., the cosine) between the phrase vectors of consecutive phrases, or phrases separated by another intervening phrase.

To complement the semantic analysis, we defined another measure for processing the documents, on the basis of Part Of Speech tagging (POS-Tag). This consists of labeling every word by its grammatical function. For example, the sentence ‘The cat is under the table’ is tagged by the POS-Tag procedure as (('The', 'DT'), ('cat', 'NN'), ('is', 'VBZ'), ('under', 'IN'), ('the', 'DT'), ('table', 'NN')) where DT is the tag for determiners, NN for nouns, VBZ for verbs, and IN for prepositions. For every transcript, we calculated the POS-Tag information (with NLTK^5^) and used the frequencies of each tag as an additional attribute of the text. Tagging automation uses a hand-tagged corpus to train a parsing process using a variety of heuristics. NLTK uses a model called Pen Tree Bank.

#### Code availability

Code for speech preprocessing (WordNet lemmatizer) and POS-Tag (Pen Tree Bank) is available open access through the NLTK (http://www.nltk.org/).^5^

#### Classification

**Figure 2:**
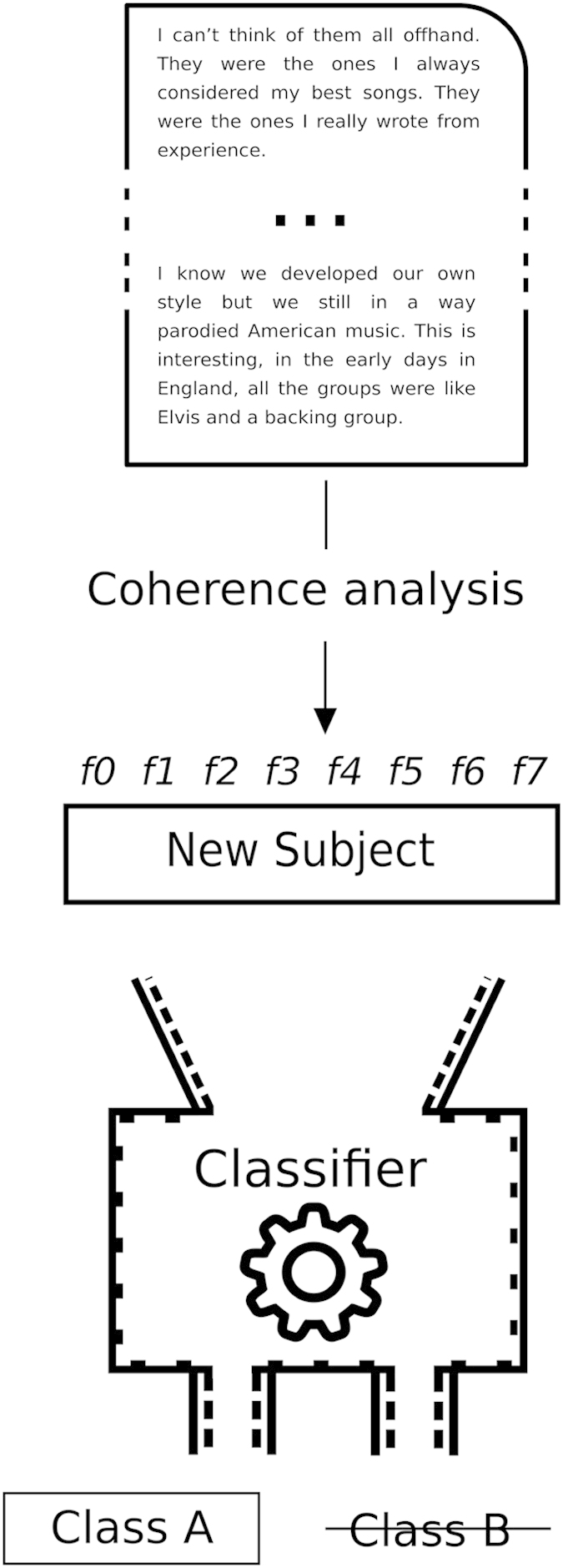
Pipeline for cross-validation of the Machine Learning classifier. A vector of features for each participant is extracted and fed into the classifier that was trained on the other participants’ data. The classifier is used to predict outcome for the left-out, or test, participant. Each participant is sequentially left out of the training data set to serve as the test subject once, resulting in accuracy of prediction data for all participants.

A cross-validated classifier is a Machine Learning algorithm with two stages: in the first stage, it learns the underlying patterns of the data using a subset of samples. The learned model is used in the second stage to predict the labels of samples not used during the learning stage (Figure 2).

We used features derived from the semantic coherence analyses and the POS-Tag extraction, providing a vector of features for each participant's text. With this information, we trained the classifier to learn the features that discriminated among participants who did not subsequently develop psychosis (CHR−) from the group who did (CHR+).

The convex hull of a set of points is the minimal convex polyhedron that contains them. A convex hull classifier was implemented as follows: during training, we sequentially excluded one CHR+ or CHR− participant to be used for testing (leave-one-subject-out cross-validation). Using the training labels, we computed the convex hull of the CHR− set, and then tested whether the left-out sample was inside the hull (predicting CHR−) or outside (predicting CHR+). Each individual was sequentially excluded from the training set used to compute the convex hull to serve as the test subject, providing accuracy of prediction data for all participants.

The semantic coherence feature that best contributed to classification of subsequent psychosis onset was the minimum coherence between two consecutive phrases (i.e., the maximum discontinuity) that occurred in the interview. The syntactic measure included in classification was the frequency of use of determiners (‘that’, ‘what’, ‘whatever’, ‘which’, and ‘whichever’), normalized by the phrase length. Because speech in emergent psychosis often shows marked reductions in verbosity (referred to clinically as poverty of speech), we also included the maximum number of words per phrase in the classification.

#### Validation

To further probe findings from the CHR analyses, we also conducted the following validation analyses:

##### Does the coherence measure index ‘disorder’ in a text?

Because the concept of semantic coherence we employed does not have a mathematical definition, in this validation we tested the coherence measure against a corpus of classic literature and assessed how the measure changed when we modified the original texts in a way that is relevant to the concept of semantic coherence.

On the basis of the hypothesis that a text that makes sense will produce a high coherence score, we applied different levels of ‘disorder’ to a range of texts to determine whether the method could detect these modifications. We defined each level of ‘disorder’ as the percent of the text that was moved from its original location. For example, a disorder level of 40% indicates that 4 of 10 sentences were moved and thus were no longer in their original position in the text. For each of 10 disorder levels (10–100%), we created 1,000 samples, randomly shuffling the order of the appropriate proportion of sentences. We performed coherence analysis on randomly selected chapters of the following six classic books: *On the Origin of Species* by Charles Darwin*, A Study in Scarlet* by Arthur Conan Doyle*, Moby Dick; Or, The Whale* by Herman Melville*, Pride and Prejudice* by Jane Austen*, The Adventures of Tom Sawyer* by Mark Twain, and *The Count of Monte Cristo* by Alexandre Dumas.

##### Are the speech features associated with symptoms assessed with standard diagnostic instruments?

To assess the extent to which the text features that best predicted clinical status at follow-up in CHR patients (minimum first-order coherence, density of determiners, and maximum phrase length) carry information with respect to standard clinical prodromal ratings, we computed the canonical correlation between these three text features (semantic coherence, phrase length and use of determiner pronouns) and two symptom measures on the SIPS/SOPS (total positive symptoms and total negative symptoms). The canonical correlation between two sets of features from the same samples, *X* and *Y*, estimates the linear combination of *X* features such that this combined feature has the highest correlation with an also estimated linear combination of *Y* features.

##### Ethics statement

The Institutional Review Board at the New York State Psychiatric Institute at Columbia approved these experiments, and informed consent was obtained for all subjects (parental consent with assent for minors).

## Results

### CHR analysis

Of the 34 participants, 5 were known to develop schizophrenia (or schizoaffective disorder) within 2.5 years. Respectively, their times to psychosis onset from time of speech sampling were 3, 4, 8, 12, and 16 months. Twenty-nine participants were known to not develop psychosis over follow-up, with 22 of these participants followed for 2.5 years, 4 participants followed for 2 years, and 3 followed for 1.5 years (these participants’ CHR status was ascertained closer to the end of the overall study). An additional participant’s transcript was not included in speech analyses because her clinical outcome was indeterminate; she remained psychosis-free over 1.5 years of follow-up, but may have subsequently developed psychosis after the study.

**Figure 3:**
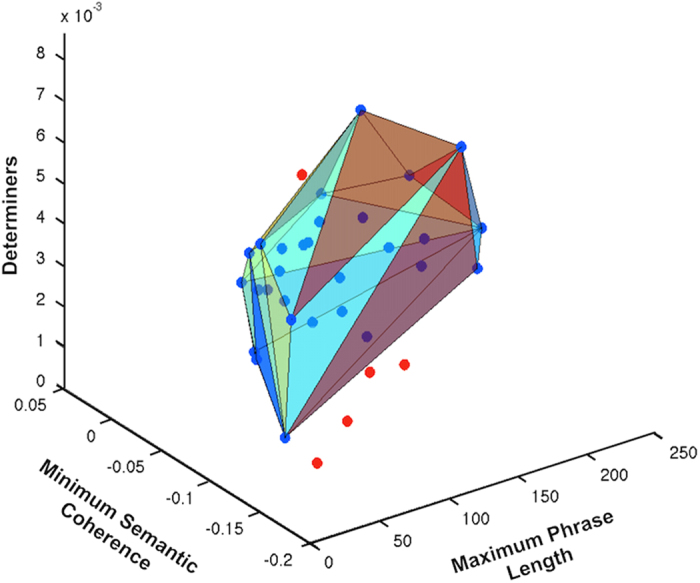
Discrimination between individuals who transitioned to psychosis (clinical high risk+ (CHR+); in red) and those who did not (CHR−; in blue) presented as the convex hull of CHR− individuals. Color shading within the convex hull is used only to illustrate volume. Discrimination was based on three features extracted from free speech using automated methods. The frequency of use of determiners (‘that’, ‘what’, ‘whatever’, ‘which’, and ‘whichever’) normalized by phrase length; the minimum semantic coherence between two consecutive phrases within the interview; and the maximum phrase length.

A graphical representation of the differentiation obtained between CHR+ and CHR− individuals using the three parameters of minimum semantic coherence, normalized use of determiners, and maximum phrase length is presented as the convex hull of the set of CHR− individuals (the minimal convex polyhedron that contains all data points) in Figure 3. The convex hull of CHR− individuals does not include any CHR+ individuals.

The convex hull classifier yielded 100% accuracy for prediction of psychosis onset. Null hypothesis tests were used to estimate the probability of obtaining this result by chance. We first partitioned the data set (*N*=34) randomly, assigning five subjects to the CHR+ label and the remainder to the CHR− group. Because some assignments for this initial test included the actual CHR+ individuals, we implemented a second test by repeating the previous scheme, including only CHR− individuals. That is, using the CHR− set, we randomly assigned CHR+ labels to 5 CHR− individuals, and estimated the probability that they would all fall outside the remaining 24 individuals randomly labeled as CHR−. Finally, we repeated the same scheme by assigning random labels to the 29 CHR− individuals (matching the original number of labels), and also randomly assigning the semantic and syntactic speech features, drawing values from a Gaussian distribution with the same mean and s.d. as the actual values. In each scheme, the probability that all five individuals labeled as CHR+ would fall outside the convex hull of CHR− individuals was less than chance, i.e., *P*<0.05.

To investigate whether standard clinical ratings could differentiate CHR+ and CHR− individuals, we entered variables from clinical ratings—the SIPS/SOPS^13^—into several classifiers. The best prediction obtained was less accurate than the automated analysis, misclassifying 3 of 5 CHR+ patients and 4 of 29 CHR− patients to yield an accuracy of 79%, consistent with prior studies (see Table 2 for classification performance metrics).

### Validations

#### The coherence measure as an index of ‘disorder’ in texts

**Figure 4:**
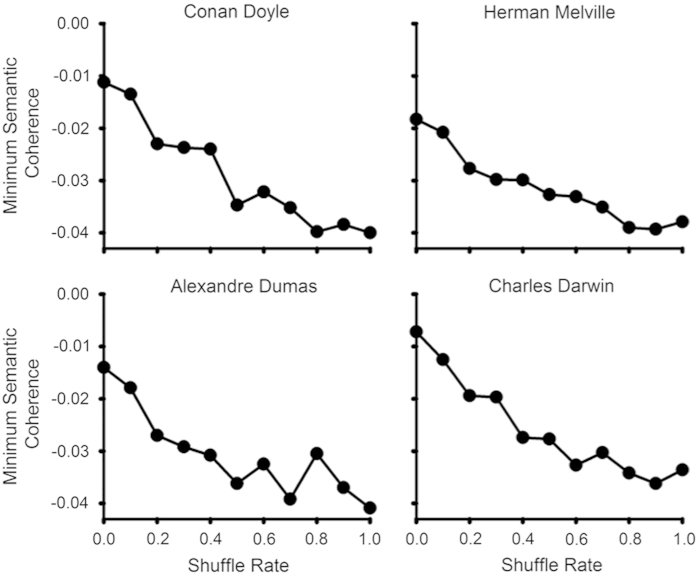
Effect of randomly shuffling a proportion of classic literary texts (degree of ‘disorder’) on the measure of semantic coherence developed. Data points represent the minimum semantic distance between two adjacent sentences within a text. Increasing levels of ‘disorder’ were associated with a decrease in the coherence measure employed.

We found that two features of the semantic coherence distributions, the minimum semantic distance for first-order coherence (i.e., the minimum coherence or maximum discontinuity between two adjacent sentences within the text sampled), and the mean semantic distance for first-order coherence (i.e., the average coherence between adjacent sentences within the text) were negatively correlated with the disorder level we produced in texts, indicating that higher levels of disorder within the text produced lower coherence scores (see Figure 4).

#### Associations between speech features and symptoms assessed with standard diagnostic instruments

**Figure 5:**
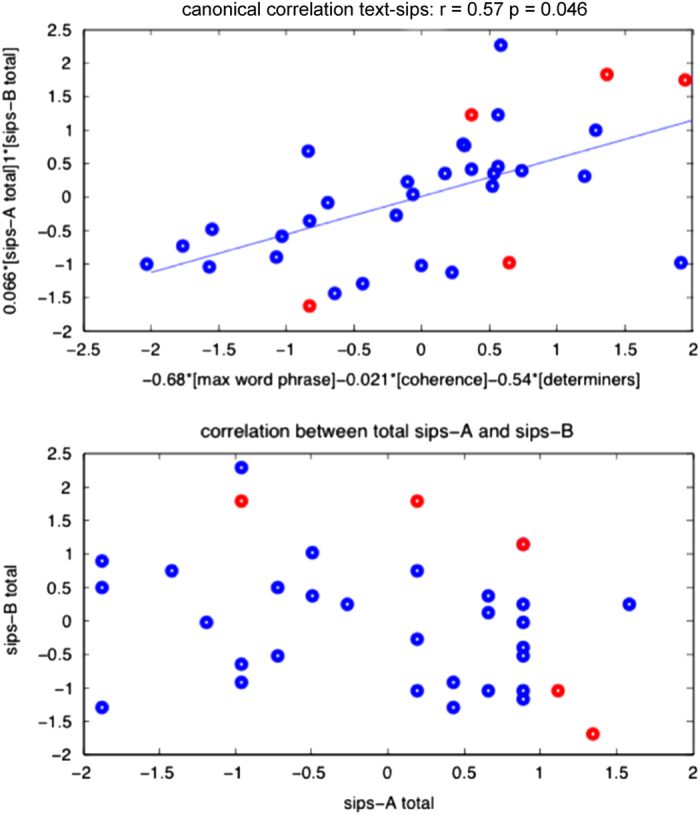
Correlations of text features and clinical ratings (top panel) and between positive (sips-A) and negative (sips-B) symptoms (bottom panel). Upper panel: canonical correlation between the text features, and the Structured Interview for Prodromal Syndromes (SIPS) features. Lower panel: scatter-plot of *A*_total_ and *B*_total_ shows no association between subthreshold psychotic symptoms and negative symptoms. In both panels, clinical high risk− (CHR−) and CHR+ are labeled with blue and red dots, respectively. For the analysis, all features were centered and normalized.

The canonical correlation analysis of text features versus the entire set of clinical prodromal features did not yield any significant correlation; however, restricting the analyses to the sums of subthreshold psychotic and negative symptom severity ratings (i.e., *A*_total_, *B*_total_) yields a correlation of *r*=0.57 and *P*=0.046, for the variables *s* (symptoms) and *t* (speech variables; Figure 5):

$$s=0.066\times A_{\mathrm{total}}+B_{\mathrm{total}},t=-0.68\times \max\;(\mathrm{wordsperphrase})-0.02\times \mathrm{coherence}-0.54\times \mathrm{determiners}.$$

In this equation, there are two symptom variables (sums of subthreshold psychotic and negative symptoms, respectively, *A*_total_, *B*_*t*otal_) and three speech variables (minimum semantic coherence, normalized use of determiners, and maximum phrase length).

That is, this analysis reveals that there is a significant correlation between *B*_total_ (i.e., sum of negative symptoms) and a combination of the maximum number of words per phrase and density of determiners. This is consistent with the concept of paucity of speech constituting a negative symptom in schizophrenia.

Finally, we observed that a scatter-plot of *A*_total_ and *B*_total_ shows a distribution reminiscent of what we find with text features: CHR+ samples tend to occupy a region outside the distribution of the CHR− set, similar to what we observe with the speech features (although less precise in terms of class separation).

Thus, although the classification based on the speech coherence analyses clearly outperformed that based on the SIPS/SOPS clinical ratings, these additional analyses indicate that the coherence features extracted are tapping dimensions that are relevant for clinical symptomatology, as measured with standardized rating scales.

## Discussion

In this initial, proof-of-principle study using a novel combination of automated semantic and syntactic speech analyses, we found that speech recorded and transcribed at baseline could accurately predict subsequent transition to psychosis in a clinical high-risk cohort. Moreover, classification based on automated analysis outperformed that based on clinical ratings, indicating that automated speech analysis can increase predictive power beyond expert clinical opinion.

Of note, the sample size employed in this initial study was small, with five participants developing psychosis during the follow-up period. This limitation meant that we were unable to divide participants into separate training and test samples, instead using cross-validation procedures to assess the predictive algorithm. This approach, although providing important information about the potential predictive capacity of these novel speech measures, may have resulted in higher estimates of the predictive accuracy of the model than would be obtained in a larger, separate sample. Thus, replication in a larger sample will be an important future research direction.

Our findings from this proof-of-concept study, although needing to be replicated in larger samples, have several implications. First, reliable identification of individuals likely to progress to schizophrenia would greatly facilitate targeted early intervention. Second, automated speech assessment, if further validated, could provide previously unavailable information for clinicians on which to base treatment and prognostic decisions, effectively functioning as a ‘laboratory test’ for psychiatry. The ease of speech recording makes this approach particularly suitable for clinical applications. Self-report of symptoms, on which much of psychiatric assessment relies, depends on the patient’s motivation and capacity to accurately report their introspective experiences, which may be influenced by psychiatric illness. Although clinicians routinely detect disorganized speech on the basis of clinical observations, our data suggest that automated analytic methods allow for superior assessment. As a direct, objective measure, automated speech analysis could thus provide important information to complement existing methods for patient assessment. Finally, these findings support the use of advanced computational methods to characterize complex human behaviors such as speech in both normal and pathological states. Such a fine-grained behavioral analysis could allow tighter mapping between psychiatrically relevant phenotypes and their underlying biology, in essence carving nature more closely at its joints. Better mapping between the behavioral and the biological is likely to lead to greater understanding of the pathophysiology of schizophrenia and other psychiatric disorders, potentially also informing psychiatric nosology.

These findings represent the initial stages in the use of emerging computer science behavioral analysis techniques, already prominently used in industry, to characterize and predict human behavior in the context of psychiatric health and illness. Using automated approaches, we were able to extract indices of speech-semantic coherence and syntax and use these to accurately predict the subsequent development of psychosis in high-risk youths. Prognostic prediction using this approach outperformed prediction on the basis of standard psychiatric ratings. Computerized analysis of complex human behaviors such as speech may present an opportunity to move psychiatry beyond reliance on self-report and clinical observation toward more objective measures of health and illness in the individual patient.

## Figures and Tables

**Table 1:** Demographics

	*CHR+ (*N*=5)*	*CHR− (*N*=29)*
Age (in years)	22.2 (3.4)	21.2 (3.6)
Gender (% male)	80%	66%
Race (% Caucasian)	40%	38%
Medications prescribed (antipsychotics and/or antidepressants)	20%	21%

Abbreviations: CHR+, clinical high-risk participants who transitioned to psychosis during follow-up; CHR−, clinical high-risk participants who did not transition to psychosis during follow-up.

**Table 2:** Classification performance metrics

*Classification*	*PPV*	*NPV*	*Sens.*	*Spec.*	*ROC*
Convex Hull 3-feature	100	100	100	100	1.00
SIPS/SOPS	33	89	40	86	0.47

Abbreviations: NPV, negative predictive value; PPV, positive predictive value; ROC, receiver operating characteristic area under the curve; Sens, sensitivity; SIPS/SOPS, classification based on baseline scores on the Structured Interview for Prodromal Syndromes/Scale for Prodromal Symptoms; Spec, specificity.

## References

[bib1] Insel TR , Landis SC . Twenty-five years of progress: the view from NIMH and NINDS. Neuron 2013; 80: 561–567. 24183009 10.1016/j.neuron.2013.09.041 PMC3859529

[bib2] Adomavicius G , Tuzhilin A . Using data mining methods to build customer profiles. IEEE Comput 2001; 34: 74–82.

[bib3] Elvevag B , Foltz PW , Weinberger DR , Goldberg TE . Quantifying incoherence in speech: an automated methodology and novel application to schizophrenia. Schizophr Res 2007; 93: 304–316. 17433866 10.1016/j.schres.2007.03.001 PMC1995127

[bib4] Poulin C , Shiner B , Thompson P , Vepstas L , Young-Xu Y , Goertzel B et al. Predicting the risk of suicide by analyzing the text of clinical notes. PLoS One 2014; 9: e85733. 24489669 10.1371/journal.pone.0085733 PMC3904866

[bib5] Bird S , Klein E , Loper E . Natural Language Processing with Python. O'Reilly Media: Sebastopol, CA, USA, 2009.

[bib6] Bedi G , Cecchi GA , Slezak DF , Carrillo F , Sigman M , de Wit H . A window into the intoxicated mind? Speech as an index of psychoactive drug effects. Neuropsychopharmacology 2014; 39: 2340–2348. 24694926 10.1038/npp.2014.80 PMC4138742

[bib7] Adler CM , Malhotra AK , Elman I , Goldberg T , Egan M , Pickar D et al. Comparison of ketamine-induced thought disorder in healthy volunteers and thought disorder in schizophrenia. Am J Psychiatry 1999; 156: 1646–1649. 10518181 10.1176/ajp.156.10.1646

[bib8] Mota NB , Vasconcelos NA , Lemos N , Pieretti AC , Kinouchi O , Cecchi GA et al. Speech graphs provide a quantitative measure of thought disorder in psychosis. PLoS One 2012; 7: e34928. 22506057 10.1371/journal.pone.0034928 PMC3322168

[bib9] Elvevag B , Foltz PW , Rosenstein M , Delisi LE . An automated method to analyze language use in patients with schizophrenia and their first-degree relatives. J Neurolinguistics 2010; 23: 270–284. 20383310 10.1016/j.jneuroling.2009.05.002 PMC2850213

[bib10] DeVylder JE , Muchomba FM , Gill KE , Ben-David S , Walder DJ , Malaspina D et al. Symptom trajectories and psychosis onset in a clinical high-risk cohort: the relevance of subthreshold thought disorder. Schizophr Res 2014; 159: 278–283. 25242361 10.1016/j.schres.2014.08.008 PMC4254175

[bib11] Gooding DC , Ott SL , Roberts SA , Erlenmeyer-Kimling L . Thought disorder in mid-childhood as a predictor of adulthood diagnostic outcome: findings from the New York High-Risk Project. Psychol Med 2013; 43: 1003–1012. 22932128 10.1017/S0033291712001791

[bib12] McGorry P , Purcell R . Youth mental health reform and early intervention: encouraging early signs. Early Interv Psychiatry 2009; 3: 161–162. 22640378 10.1111/j.1751-7893.2009.00128.x

[bib13] Miller TJ , McGlashan TH , Rosen JL , Cadenhead K , Cannon T , Ventura J et al. Prodromal assessment with the structured interview for prodromal syndromes and the scale of prodromal symptoms: predictive validity, interrater reliability, and training to reliability. Schizophr Bull 2003; 29: 703–715. 14989408 10.1093/oxfordjournals.schbul.a007040

[bib14] Holshausen K , Harvey PD , Elvevag B , Foltz PW , Bowie CR . Latent semantic variables are associated with formal thought disorder and adaptive behavior in older inpatients with schizophrenia. Cortex 2013; 55: 88–96. 23510635 10.1016/j.cortex.2013.02.006 PMC3700559

[bib15] Davidson L . Phenomenological research in schizophrenia: From philosophical anthropology to empirical science. J Phenomenol Psychol 2004; 25: 104–130.

[bib16] Ben-David S , Birnbaum M , Eilenberg M , DeVylder J , Gill K , Schienle J et al. The subjective experience of youths at clinical high risk for psychosis: a qualitative study. Psychiatr Serv 2014; 65: 1499–1501. 25179420 10.1176/appi.ps.201300527 PMC4358875

[bib17] Landauer TK , Dumais ST . A solution to Plato's problem: the latent semantic analysis theory of acquisition, induction, and representation of knowledge. Psychol Rev 1997; 104: 211–240.

